# Supplementary material for: Breeding for Virus Resistance and Its Effects on Deformed Wing Virus Infection Patterns in Honey Bee Queens
Source: Viruses. 2021 Jun 4;13(6):1074. doi: 10.3390/v13061074 (PMC8228329; doi:10.3390/v13061074)
Supplement: Supplementary file 1 [file viruses-13-01074-s001.zip › viruses-1238315-supplementary.pdf]

viruses

MDPI

Supplementary Information

Breeding for Virus Resistance and its Effects on Deformed Wing Virus Infection Patterns in Honey Bee Queens.

David Claeys Bouuaert *, Lina De Smet and Dirk C. de Graaf

Laboratory of Molecular Entomology and Bee Pathology, Ghent University, Krijgslaan 281, B-9000 Ghent, Belgium; lina.desmet@ugent.be (L.D.S.); Dirk.deGraaf@UGent.be (D.C.d.G.)
* Correspondence: david.claeysbouuaert@ugent.be

Citation: Bouuaert, D.C.; De Smet, L.; de Graaf, D.C. Breeding for Virus Resistance and Its Effects on Deformed Wing Virus Infection Patterns in Honey Bee Queens. *Viruses* **2021**, *13*, 1074. https://doi.org/10.3390/v13061074

Academic Editor: Miguel López-Ferber

Received: 12 May 2021
Accepted: 28 May 2021
Published: 4 June 2021

Publisher's Note: MDPI stays neutral with regard to jurisdictional claims in published maps and institutional affiliations.

Copyright: © 2021 by the authors. Licensee MDPI, Basel, Switzerland. This article is an open access article distributed under the terms and conditions of the Creative Commons Attribution (CC BY) license (http://creativecommons.org/licenses/by/4.0/).

Table S1. Primer sequences.

Virus	Primers	Sequence '5-'3	Size (bp)	Ref
DWV-A	DWV-F	5′-TTC ATT AAA GCC ACC TGG AAC ATC-3′	136	[55]
	DWV-R	5′-TTT CCT CAT TAA CTG TGT CGT TGA-3′		
DWV-B	VDV-F2	5′-TAT CTT CAT TAA AAC CGC CAG GCT-3′	140	[56]
	VDV-R2a	5′-CTT CCT CAT TAA CTG AGT TGT TGT C-3′		

Table S2. overview of number of infections and infection loads.

	Status	Eviscerated Abdomen		Guts		Head		Ovaries		Spermatheca		Thorax	
		Nr. of infections	Avg. infection load	Nr. of infections	Avg. infection load	Nr. of infections	Avg. infection load	Nr. of infections	Avg. infection load	Nr. of infections	Avg. infection load	Nr. of infections	Avg. infection load
DWV-A	QDS-	13	3.9	13	5.2	11	3.5	15	5.5	16	4.1	13	3.4
	QDS+	3	3.1	12	4.3	2	3.4	13	4.9	12	3.1	3	3.1
DWV-B	QDS-	17	4.2	19	3.8	2	3.0	19	4.5	17	3.3	3	2.5
	QDS+	15	3.1	19	3.1	0	-	20	4.6	17	4.4	0	-
